# Glycolipid Binding Preferences of Shiga Toxin Variants

**DOI:** 10.1371/journal.pone.0101173

**Published:** 2014-07-01

**Authors:** Sayali S. Karve, Alison A. Weiss

**Affiliations:** Department of Molecular Genetics, Biochemistry and Microbiology, University of Cincinnati, Cincinnati, Ohio, United States of America; Columbia University, United States of America

## Abstract

The major virulence factor of Shiga toxin producing *E. coli*, is Shiga toxin (Stx), an AB_5_ toxin that consists of a ribosomal RNA-cleaving A-subunit surrounded by a pentamer of receptor-binding B subunits. The two major isoforms, Stx1 and Stx2, and Stx2 variants (Stx2a-h) significantly differ in toxicity. The exact reason for this toxicity difference is unknown, however different receptor binding preferences are speculated to play a role. Previous studies used enzyme linked immunosorbent assay (ELISA) to study binding of Stx1 and Stx2a toxoids to glycolipid receptors. Here, we studied binding of holotoxin and B-subunits of Stx1, Stx2a, Stx2b, Stx2c and Stx2d to glycolipid receptors globotriaosylceramide (Gb3) and globotetraosylceramide (Gb4) in the presence of cell membrane components such as phosphatidylcholine (PC), cholesterol (Ch) and other neutral glycolipids. In the absence of PC and Ch, holotoxins of Stx2 variants bound to mixtures of Gb3 with other glycolipids but not to Gb3 or Gb4 alone. Binding of all Stx holotoxins significantly increased in the presence of PC and Ch. Previously, Stx2a has been shown to form a less stable B-pentamer compared to Stx1. However, its effect on glycolipid receptor binding is unknown. In this study, we showed that even in the absence of the A-subunit, the B-subunits of both Stx1 and Stx2a were able to bind to the glycolipids and the more stable B-pentamer formed by Stx1 bound better than the less stable pentamer of Stx2a. B-subunit mutant of Stx1 L41Q, which shows similar stability as Stx2a B-subunits, lacked glycolipid binding, suggesting that pentamerization is more critical for binding of Stx1 than Stx2a.

## Introduction

Shiga toxin producing *E. coli* (STEC) [1], including serogroups O157:H7 and non-O157, are one of the leading causes of food poisoning worldwide [2]. Ingestion of as few as 30 bacteria is enough to produce disease symptoms [3]. STEC infections result in a range of symptoms from mild diarrhea to hemorrhagic colitis [4], [5]. About 10% of the infected progress to the life-threatening kidney disorder called as hemolytic uremic syndrome (HUS) [6]–[12]. Currently there is no specific treatment for HUS and conventional antibiotic treatment is known to worsen HUS symptoms [13].

**Figure 1:**
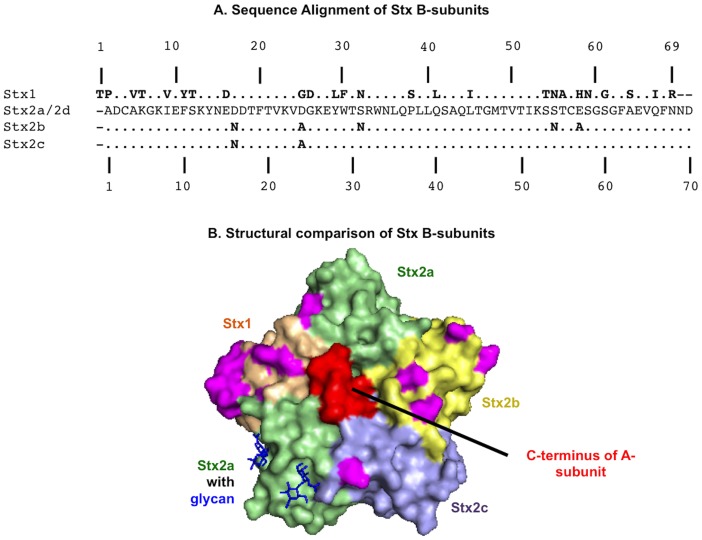
Comparison of B-subunits of Stx variants: (A) Amino acid sequence comparison. Amino acid sequences of Stx B-subunits were aligned using BLASTP (NCBI/BLAST). Periods indicate identity and dashes indicate absent amino acids. Amino acid differences with respect to Stx2a are denoted in bold. Numbering starts with the first amino acid of the mature peptide. **(B) Structural comparison.** The mutagenesis function of PYMOL was used to substitute amino acids of the Stx variants into the crystal structure of disaccharide bound Stx2a (PDB: 4M1U). The structures are oriented to display the receptor binding face of the B-subunits, with an individual subunit representing a different Stx variant. Color-coding is as follows: wheat, Stx1; green, Stx2a; yellow, Stx2b, blue, Stx2c, blue, bound disaccharide; red, A-tail of Stx2a; pink, amino acid polymorphisms with respect to Stx2a. Note that the Stx B-pentamer is made up of identical B-subunits.

The primary virulence factor of STEC is Shiga toxin (Stx), which belongs to the AB_5_ group of toxins [14], [15]. The A-subunit is responsible for inhibiting protein synthesis of the target cells by cleaving the N-glycosidic bond of adenine 4324 in 28S rRNA and preventing tRNA binding [16]. The A-subunit is non-covalently attached to a pentamer of identical B-subunits, which bind to host cell surface receptors mediating cytoplasmic delivery of the A-subunit [17]–[20]. Stx includes two immunologically distinct isoforms, Stx1 and Stx2, which share about 60% amino acid identity and a highly conserved general structure. Stx2 is further subtyped into 8 variants (Stx2a-Stx2h), which display approximately 90% amino acid identity (**Figure 1**). In spite of the high structural similarity, these variants significantly differ in toxicity, with Stx2a being over 100-fold more toxic to mice than Stx1, and variant isoform Stx2b [21]–[26]. STEC strains can express one or more Stx variants. However, strains producing Stx2a, Stx2c and Stx2d are more commonly associated with HUS in humans than those producing Stx1 or Stx2b [27]. Previously, in cell free in-vitro translation inhibition assays A-subunits of Stx variants displayed similar activities [28]. This suggested that the enzymatic activities of A-subunits are not likely responsible for the toxicity differences between Stx variants. On the contrary, Stx B-subunits have been shown to display differences in receptor recognition, and influence cellular toxicity [27]–[32].

The B-subunits of Stx recognize cell surface glycolipid globotriaosylceramide (Gb3) [33] and to a lesser extent globotetraosylceramide (Gb4) as receptors [27], [34] (**Table 1**). Gb3 is composed of a tri-saccharide (Galα1-4Galβ1-4Glc), called Pk trisaccharide, which is attached to the lipid, ceramide. Gb4 is derived from Gb3, and is composed of a tetra-saccharide (GalNAcβ1-3Galα1-4Galβ1-4Glc), called P trisaccharide, which is also attached to ceramide. These glycolipids are generally located in phosphatidyl choline (PC)- and cholesterol (Ch)-rich cell membrane microdomains called lipid rafts [35]–[39].

**Table 1:** Glycolipids used in this study.

Name (Abbreviation)	Structure	Formula
Globotriaosylceramide (Gb3)	Galα[1]–[4]Galβ[1]–[4]Glc-Ceramide	C_60_H_113_NO_18_
Globotetraosylceramide (Gb4)	GalNAcβ[1]–[3]Galα[1]–[4]Galβ[1]–[4]Glc-Ceramide	C_68_H_126_N_2_O_23_
Galactosylceramide (Gal-cer)	Gal-ceramide	C_48_H_93_NO_9_
Lactosylceramide (Lac-cer)	Galβ[1]–[4]Glc-Ceramide	C_53_H_101_NO_13_
Glucosylceramide (Glc-cer)	Glc-ceramide	C_46_H_89_NO_8_

Previous studies examined binding of purified Stx1 and Stx2a to the neutral glycolipids, alone or in mixtures and each variant displayed a unique binding profile [40]. Similarly, differences in receptor recognition of Stx2 variants are known to mediate host specificity. Stx2a, associated with human disease, prefers binding to Gb3, while Stx2e, associated with swine disease, prefers Gb4 [41]. Glycolipid-binding sites and preferences of highly toxic Stx2 variants including Stx2c and Stx2d, or weakly toxic variants Stx2b have not yet been reported.

Crystal structure of Stx1 B-subunit with the Pk trisaccharide has been determined. It indicates the presence of three Pk binding sites per B-monomer, for a total of approximately 15 Pk-binding sites per B-pentamer [42]. The affinity of an individual binding site for its glycan receptor is very weak [32], [43], and tight binding is achieved by avidity, or the ability to simultaneously engage multiple receptor binding sites. Recently, Jacobson et al published the crystal structure of Stx2a holotoxin bound to a Pk derivative, NHAc-Pk. Only two sites on the B-pentamer displayed density for NHAc-Pk, [44], suggesting that Stx1 and Stx2a significantly differ in their receptor recognition as well as the number of potential binding sites.

While avidity is necessary for high affinity receptor binding, paradoxically studies using analytical centrifugation (AUC), mass spectrometry and circular dichroism indicate that B-subunits of Stx1 and Stx2a differ in their abilities to form a stable pentamer [45], [46]. Conrady *et al* identified a glutamine (Q40) in Stx2a within an otherwise hydrophobic B-subunit interface. The corresponding amino acid in Stx1 was a hydrophobic leucine (L41). Interchanging these residues (Stx1-L41Q and Stx2a-Q40L) reversed the stability phenotypes of Stx1 and Stx2a. Interestingly, the destabilizing amino acid, Q40 is conserved among all Stx2 variants (**Figure 1**), suggesting that destabilization of the B-pentamer might impart a selective advantage to Stx2. The physiological significance of the differences in B-pentamer stabilities is currently unclear.

In this study, using enzyme linked immunosorbent assay (ELISA) we showed that holotoxins and B-subunits of Stx variants display distinct glycolipid binding profiles. In addition, we determined that stabilities of the B-subunits are important determinants of glycolipid binding affinities. Taken together, this report gives information about receptor preferences of Stx variants and the role of B-subunits in these receptor interactions.

## Materials and Methods

### Glycolipids and other lipids

The glycolipids used in this study were purchased from Matreya Inc. (Pleasant Gap, PA) and have been enlisted in **Table 1**.

### Antibodies

Rabbit polyclonal antibodies against Stx1 A-subunit and Stx2 A-subunit were obtained from Meridian Bioscience. Mouse monoclonal antibodies against Stx1 A-subunit and Stx2 A-subunit were obtained from Biodefense and Emerging Infections (BEI) Research Resources Repository. Mouse monoclonal antibody against Stx1 B-subunit was obtained from BEI resources. Chicken polyclonal antibody against Stx2 B-subunit was obtained from Lampart Biologicals. Peroxidase-conjugated goat anti-mouse, anti-rabbit and anti-chicken IgG's were purchased from MP Biomedicals.

### Production of Stx Holotoxin Supernatants

The Stx strains used in this study are summarized in **Table 2**. Starter cultures of Stx holotoxins were grown in Mueller-Hinton (MH) broth. Overnight starter cultures were diluted 1/100 in fresh MH broth and grown with shaking at 37°C until the optical density at 600 nm reached approximately 1. Stx expression was induced by treating the cultures with ciprofloxacin (10 ng/ml) to induce the phage lytic cycle and the cultures were shaken overnight at 37°C. The cells were subsequently removed by centrifugation and supernatants containing Stx holotoxins were filter-sterilized. Presence of both A- and B-subunits in the supernatants was confirmed by Western blots using antibodies against Stx A- and B-subunits. Vero monkey kidney cell line [47] (a gift from Alison O'Brien), transfected to express *luc2p*, a gene for destabilized luciferase [48], was used to confirm the protein synthesis inhibitory activity of the Stx supernatants.

**Table 2:** Sources of Stx-producing strains used in this study.

			Protein Accession no. (NCBI)	
Toxin	Strain	Source	A-subunit	B-subunit
**Stx1**	C600::H19B	Alison O'Brien	AAA98347	AAA98348
**Stx2a**	C600∶933W	Alison O'Brien	AAD25445	AAD25446
**Stx2b**	EH250	Statens Serum Institut	AAD12174.1	AAD12175.1
**Stx2c**	C394-03	Statens Serum Institut	ABB36584.1	ABB36585.1
**Stx2d**	3024-94	Alison O'Brien	HQ585061	HQ585062

### Toxin Quantification

Western blots were performed using crude supernatants of unknown concentration, along with purified Stx1 and Stx2a holotoxins of known concentrations. Monoclonal antibodies against Stx1 and Stx2 A-subunits were used for the Western blots. The band densities corresponding to the A-subunit of the toxins with known concentrations were recorded using ImageJ software and were considered as standards. Concentrations of the toxin supernatants were then determined by comparing their band densities with the standards using Analysis program of the ImageJ software.

### Expression and Purification of Stx B-subunits

Expression and purification of B-subunits of Stx variants was performed as previously described. Briefly, pET21b(+) expression plasmids encoding the B-subunits of Stx variants (**Table 3**) were transformed into *E. coli* BL21(DE3)pLysS (Novagen). Transformants were cultured in Luria-Bertani broth containing ampicillin (250 µg.ml-1) and chloramphenicol (34 µg.ml-1). This was followed by cold-shock induction of the Stx B-subunits with 0.1 mM IPTG and 20% ethanol at 20°C. Proteins were extracted by freeze-thaw, sonication and purified by ammonium sulfate precipitation (40–70%), Q-sepharose Fast Flow ion exchange chromatography (GE Healthcare, Uppsala, Sweden), Superdex 75 HiLoad 26/60 size exclusion chromatography (GE Healthcare) and UnoQ Q6R ion exchange chromatography (Bio-Rad, Hercules, CA). Presence of B-subunits in the preparations was confirmed by Western blot. Protein purity was verified by the presence of a single band at 8 kDa on Coomassie stained SDS-PAGE gels, corresponding to the molecular weight of a single B-subunit. Bicinchoninic Acid Protein Assay (Pierce, Rockford, IL) was used to calculate the protein concentrations.

**Table 3:** Sources of B-subunit plasmids used in this study.

Plasmids	B-subunit	Source and/or Reference
**pMFUC-20**	Stx1 B-Wild type	[2]
**pSHUC-5**	Stx1 B-L41Q	[2]
**pMFUC-21**	Stx2a B-Wind type	[2]
**pSHUC-6**	Stx2a B-Q40L	[2]
**pCF-6**	Stx2d B-Wild type	This study
**pCF-7**	Stx2c B-Wild Type	This study

### Glycolipid ELISA

We used ELISA to study equilibrium glycolipid binding of Stx holotoxin supernatants and Stx B-subunits. Stock suspensions of glycolipids, PC and Ch were made in a 1∶1 mixture of chloroform and methanol. Working mixtures of glycolipids, PC and Ch were made from the stock suspensions in the molar ratio of 1∶3∶3 respectively in methanol, as previously described [32]. 50 µl per well of single or mixed glycolipids, with or without PC and Ch were added to hydrophobic Mictotiter plates (Microfluor 1, Thermo Scientific) and allowed to dry in the fume hood overnight in order to facilitate immobilization. Wells coated with PC, Ch, PC+Ch and methanol alone, were used as the negative controls. Before starting the experiment, the plates were cooled down at 4°C for at least 1 hour. The cooled plates were blocked with 2% bovine serum albumin (BSA) in phosphate buffered saline (PBS; 8.1 mM Na_2_HPO_4_, 1.5 mM KH_2_PO_4_, 128 mM NaCl, 2.7 mM KCl), pH 7.4. Half log dilutions of Stx holotoxin supernatants or purified B-subunits were prepared in PBS and subsequently added to the wells. The plates allowed to incubate for 1 hour at 37°C. The bound proteins were then incubated with respective primary and secondary antibodies. Finally, the plates were developed with QuantaBlue fluorogenic peroxidase substrate (Pierce, Rockford, IL) and read using FL600 microplate fluorescence reader (Biotek). The plates were washed between each step with ice cold PBS containing 1% BSA and all steps were performed at 4°C, unless otherwise specified. The signal was recorded as Relative Fluorescence Units (RFU's). During analysis, the RFU's corresponding with the negative controls were subtracted from the RFU's corresponding to the proteins. Binding curves were plotted using Prism 5.0 (GraphPad software, La Jolla, CA). Statistical analyses were performed on three individual repeats.

## Results

### Glycolipid binding of Stx holotoxins

**Figure 2:**
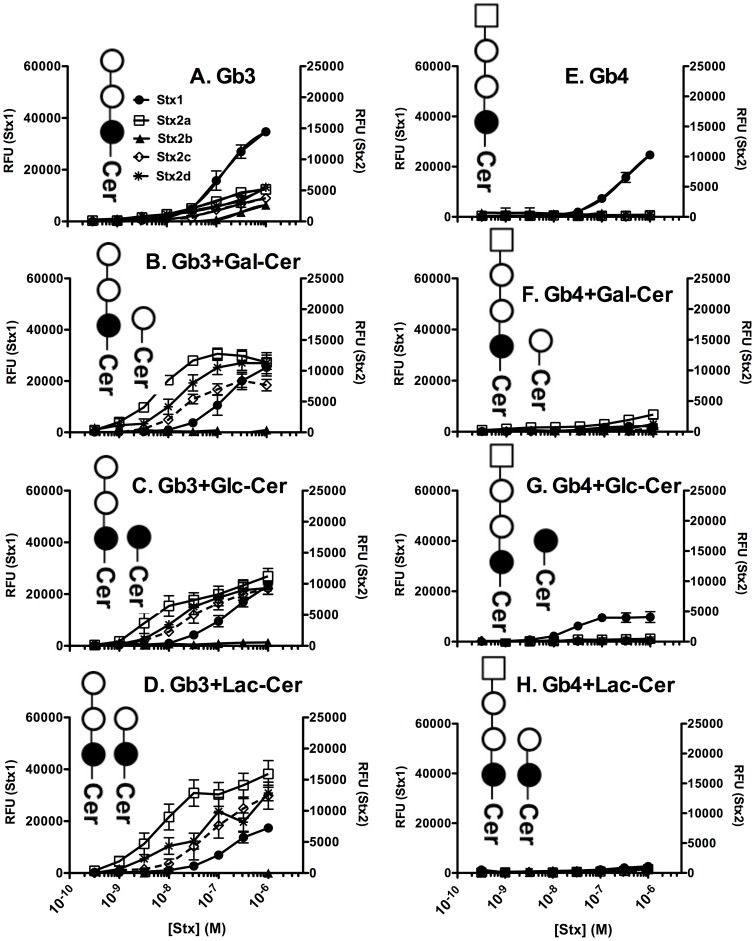
Binding of Stx holotoxins to glycolipid mixtures in absence of PC and Ch. Binding was assessed by ELISA at 37°C using serial dilutions of Stx variants. **A. Gb3; B. Gb3+Gal-Cer; C. Gb3+Glc-Cer; D. Gb3+Lac-Cer; E. Gb4; F. Gb4+Gal-Cer; Gb4+Glc-Cer; Gb4+Lac-Cer.** Mixtures of glycolipids were prepared in methanol in the ratio of 1∶1 of the two glycolipids. Total concentration of 200 ng glycolipid was added per well. As negative control toxins were incubated with plate sham-coated with methanol. In all experiments, background RFU values obtained in methanol were subtracted from each value. The RFU signal is the mean of three independent experiments and error bars indicate standard deviation (SD).

Stx1 and Stx2a display significant differences in glycolipid binding [40]; we wanted to determine if the Stx2 variants also display differences in glycolipid recognition. We used ELISA to examine binding of Stx1, Stx2a, Stx2b, Stx2c and Stx2d using combinations of Gb3, Gb4, and other neutral glycolipids, Gal-Cer, Glc-Cer, and Lac-Cer. Slight binding to Gb3 and Gb4 alone was observed (**Figures 2A and E**). Among the Stx variants tested, Stx1 showed the highest ‘maximum RFU's upon Stx binding' (Bmax) for binding to Gb3 alone (**Figure 2A**); however the dissociation constant (K_D_) values for Stx1-Gb3 and Stx2a-Gb3 were similar (**Table 4**). None of the Stx variants bound to Glc-Cer, Lac-Cer, and Gal-Cer alone (data not shown).

**Table 4:** EC_50_ values (in), glycolipid binding dissociation constants for Stx holotoxin and B-subunits (N.D.: Not determined due to insignificant binding).

	Glycolipid Binding, K_D_ in μM (Hill coefficient)				AUC[46]
	Gb3+PC+Ch	Gb4+PC+Ch	Gb3	Gb4	EC_50_ (μM)
**Holotoxin**					
**Stx1**	0.046 (1)	0.105 (1)	0.139 (1.2)	0.308 (1.2)	-
**Stx2a**	0.025 (0.7)	0.035 (0.8)	0.074 (0.8)	N.D.	-
**Stx2b**	0.094 (0.8)	N.D.	0.308 (0.7)	N.D.	-
**Stx2c**	0.210 (0.7)	0.915 (0.8)	0.192 (0.8)	N.D.	-
**Stx2d**	0.032 (0.9)	0.653 (0.9)	N.D.	N.D.	-
**B-subunits**					
**Stx1**	0.018 (1)	0.011 (1.1)	0.027 (1)	0.026 (1.1)	0.043
**Stx1-L41Q**	3.372 (1.6)	2.329 (2.1)	2.234 (2)	2.584 (2.1)	1.060
**Stx2a**	0.141 (1.6)	0.235 (1.4)	0.418 (2.7)	0.559 (2.2)	2.290
**Stx2a-Q40L**	0.003 (0.8)	0.005 (1.0)	0.005 (0.7)	0.128 (0.8)	0.693
**Stx2c**	0.583 (1.6)	0.453 (1.6)	0.117 (1.2)	0.778 (1.9)	-
**Stx2d**	0.176 (1.5)	0.243 (1.5)	0.278 (2.6)	0.423 (2)	-

Next we examined Stx binding to mixtures of Gb3 and Gb4 with Gal-Cer, Lac-Cer and Glc-Cer. Stx1 displayed dose dependent binding to 1∶1 mixtures of Gb3 with Gal-Cer (**Figure 2B**), Glc-Cer (**Figure 2C**) and Lac-Cer (**Figure 2D**). Among the Gb4 combinations, Stx1 showed weak binding to Gb4 mixed with Glc-Cer (**Figure 2G**). No significant Stx1 binding was observed for Gb4 mixed with Gal-Cer (**Figure 2F**) or Lac-Cer (**Figure 2H**).

Compared to Gb3 alone (**Figure 2A**), binding of Stx2 variants considerably increased when Gb3 was presented in a 1∶1 mixture with other glycolipids. Interestingly, Stx2a, Stx2c and Stx2d bound better than Stx1 to these Gb3 combinations (**Figures 2B–D**). Binding profiles of Stx2a, Stx2c and Stx2d were similar for Gb3+Glc-Cer (**Figure 2C**). On the other hand, Stx2a and Stx2d bound better than Stx2c to Gb3+Gal-Cer (**Figure 2B**) and Gb3+Lac-Cer (**Figure 2D**). Stx2b marginally bound to Gb3 alone and did not bind to any of the Gb3 mixtures. None of the Stx2 variants bound to Gb4 mixtures at the concentrations tested (**Figures 2F–H**).

**Figure 3:**
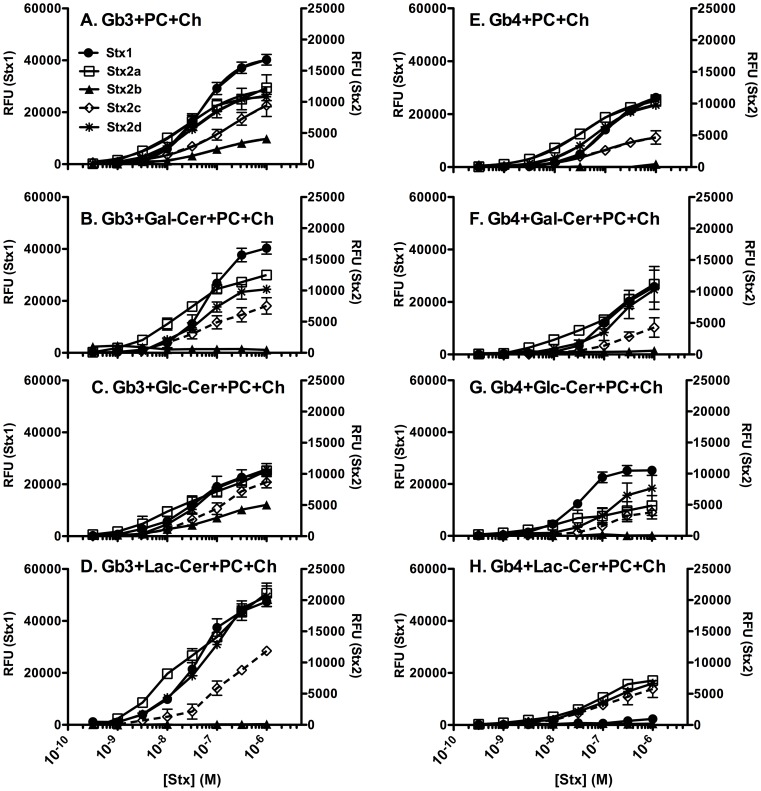
Binding of Stx holotoxins to glycolipid mixtures in presence of PC and Ch. Binding was assessed by ELISA at 37°C using serial dilutions of Stx variants. **A. Gb3; B. Gb3+Gal−Cer; C. Gb3+Glc−Cer; D. Gb3+Lac−Cer; E. Gb4; F. Gb4+Gal−Cer; Gb4+Glc−Cer; Gb4+Lac−Cer.** Mixtures of glycolipid 1, glycolipid 2, PC and Ch were prepared in ratio of 1∶1∶3∶3, respectively, to make 200 ng of total glycolipid concentration per well. Binding was assessed as described in Figure 2. The RFU signal is the mean of three independent experiments and error bars indicate SD.

Since glycolipids are generally located in the PC- and Ch-rich lipid rafts of the cell membrane, glycolipid binding of Stx was assessed in the presence of PC and Ch. Stx variants did not bind to the monosaccharide or disaccharide glycolipids, Gal-Cer, Glc-Cer or Lac-Cer even in the presence of PC and Ch (data not shown). However, presence of PC and Ch increased binding of all Stx variants to both Gb3 (**Figures 2A–D** **and** **3A–D**) and Gb4 (**Figures 2E–H** **and** **3E–H**) mixtures as seen by either decrease in K_D_ values (**Table 4**) or increase in Bmax (**Figure 3**). Stx1 bound to almost all glycolipid combinations tested; however, 1∶1 mixture of Gb4+Lac-Cer was not able to capture Stx1 even in the presence of PC and Ch (**Figure 3H**). Among the Stx2 variants, Stx2a and Stx2d showed comparable glycolipid binding profiles, followed by Stx2c. The least toxic variant Stx2b bound only to Gb3+PC+Ch and to Gb3+Glc−Cer+PC+Ch (**Figures 3A and C**). In general, at high toxin concentrations (1 µM), glycolipid binding of Stx1 was equivalent to Stx2a. However, at lower concentrations Stx2a bound better than Stx1 to most of the glycolipid combinations tested.

### Glycolipid binding of Stx B-subunits

Stx binds to the target cell surface mainly via its B-subunits and this binding is suggested to be an important step in Stx mediated toxicity [30]. As a result it is important to understand the details of B-subunit interaction with the cell surface receptors. In this study using different combinations of neutral glycolipids, we examined the glycolipid receptor interactions of Stx B-subunits.

**Figure 4:**
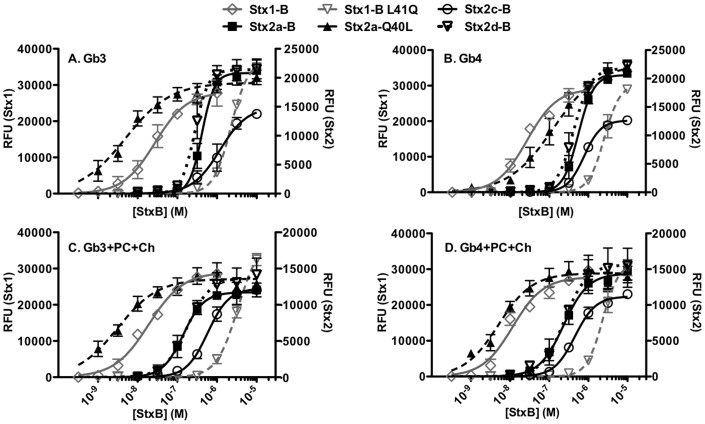
Glycolipid binding of Stx B-subunits. Serial dilutions of Stx B-subunits were titrated against immobilized glycolipids to obtain the dose response curves. **A. Gb3, B. Gb3+PC+Ch, C. Gb4, D. Gb4+PC+Ch**. B-subunits were incubated with methanol-coated wells as negative controls. Binding was assessed as described in Figure 2. The RFU signal is the mean of at least three independent experiments and error bars indicate SD. Symbols represent experimental data, while lines represent the fitted model for that data analyzed with Prism5 (GraphPad software, La Jolla, CA).

**Figure 4** shows binding of purified Stx B-subunits to Gb3 (**Figures 4A and C**) and Gb4 (**Figures 4B and D**) in presence or absence of PC and Ch. B-subunits of Stx1 displayed stronger glycolipid binding compared to Stx2 variants, as seen by a lower K_D_ for Stx1 (**Table 4**). Among the Stx2 variants, the B-subunits of Stx2a, Stx2c and Stx2d displayed similar glycolipid binding affinity. The presence of PC and Ch did not significantly change binding of the Stx B-subunits to Gb3 and Gb4. This was in contrast to the holotoxins, which preferred binding to Gb3 and Gb4 in the presence of PC and Ch.

Previous studies reported the molar concentration of B-monomer required to achieve 50% assembly (EC_50_) indicating the B-pentamer stabilities (**Table 4**) [46]. In our ELISA experiments the glycolipid binding of the B-subunits correlated with their pentamer stabilities. Binding reached saturation at the concentrations of the B-subunits above EC_50_ for pentamerization (**Figure 4**). In order to further investigate the role of pentamerization in B-subunit receptor recognition, we tested the glycolipid binding of Stx1 mutant with decreased B-pentamer stability, L41Q and Stx2a mutant with increased B-pentamer stability, Q40L. The destabilized Stx1 mutant L41Q displayed significantly reduced glycolipid affinity than wild type B-subunits of both Stx1 and Stx2a (**Figure 4** **and** **Table 4**). On the other hand, the stabilized Stx2a mutant Q40L displayed increased glycolipid affinity compared to the wild type of Stx2a (**Figure 4** and **Table 4**). Glycolipid binding profile of Q40L resembled Stx1 B-subunits.

Next we determined the Hill coefficients (h) for glycolipid binding of the B-subunits. Hill coefficients are a measure of cooperativity in binding. A Hill coefficient value of 1 indicates no cooperativity; a value of greater than 1 indicates positive cooperativity, where binding of one ligand facilitates binding of subsequent ligands; a value of less than 1 suggests negative cooperativity, where binding of one ligand suppresses the binding of subsequent ligands. The h-values for glycolipid binding of the B-subunits were significantly different. The h-value for binding of Stx1 B-subunits to the Gb3 mixture was close to 1, whereas B-subunits of Stx2a, Stx2c and Stx2d bound to glycolipids with h-values much greater than 1 (**Table 4**). Interestingly, the stability mutant of Stx2a, Q40L bound with a h-value more similar to Stx1, or around 1. On the other hand, the h-value of the destabilized mutant of Stx1, L41Q was 2.0, more similar to Stx2a.

### Role of ceramide in Stx glycolipid interaction

**Figure 5:**
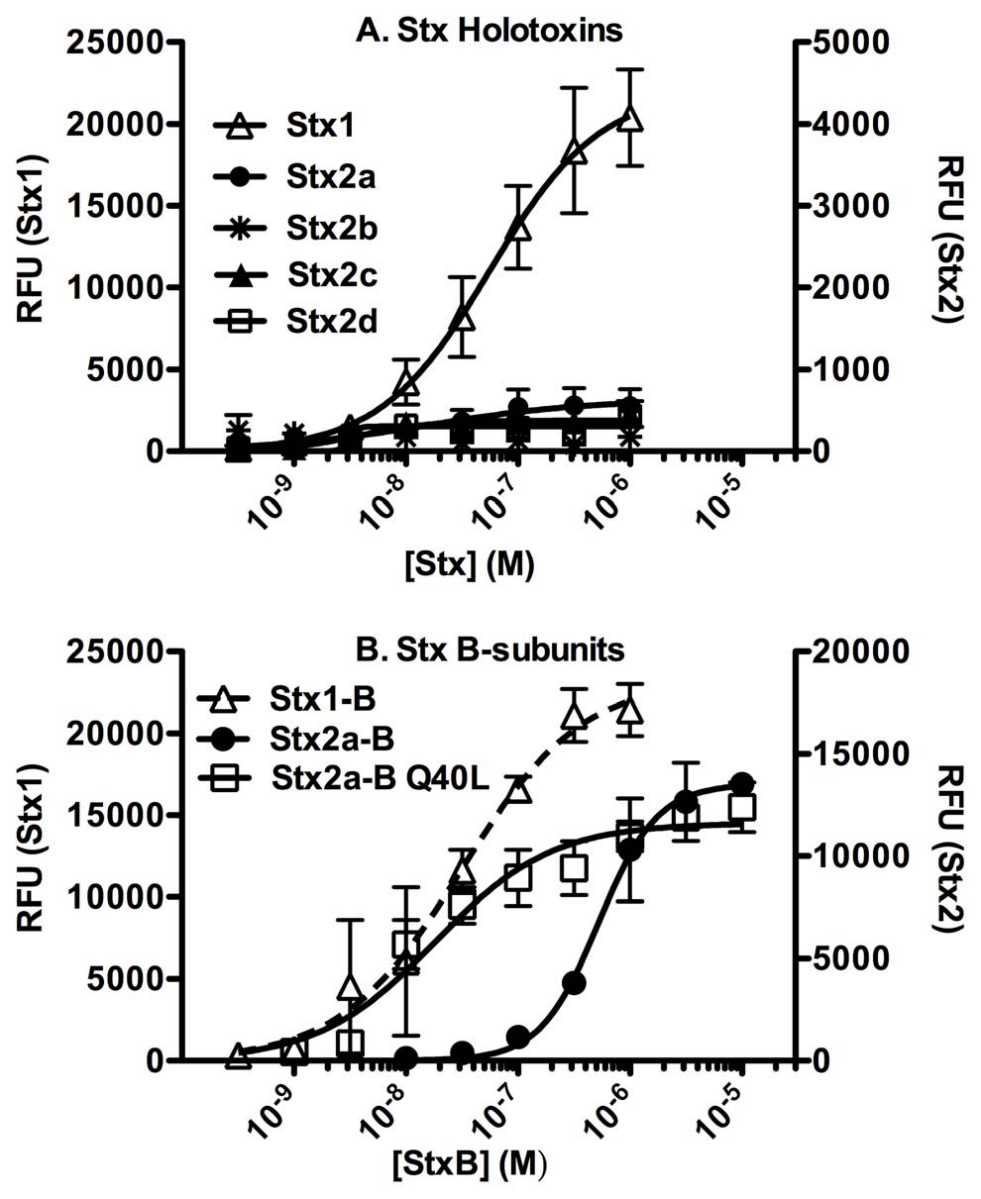
Binding of holotoxin and B-subunits to Lyso-Gb3. ELISA was used to study binding of serial dilutions of **A. Stx holotoxins** and **B. B-subunits**. Binding was assessed as described in Figure 2. The RFU signal is the mean of three independent experiments and error bars indicate SD.

Previous studies using purified toxoids showed that the ceramide portion of Gb3 is critical for binding of Stx2a; but is dispensable for binding of Stx1 [40]. We investigated the requirement of ceramide for Gb3 binding of Stx variants in the holotoxin form. Deacetylated Gb3 (Lyso-Gb3), which lacks a carbonyl and a fatty acid chain in the sphingosine of Gb3, was used. **Figure 5A** shows binding of Stx holotoxins to Lyso-Gb3 in the presence of PC and Ch by ELISA. Crude supernatant of Stx1 holotoxin displayed binding to Lyso-Gb3+PC+Ch, although it was reduced compared to binding of Stx1 to Gb3+PC+ Ch (**Figures 5A** **and** **3A**). On the other hand, similar to the toxoid, none of the Stx2 holotoxins bound to Lyso-Gb3+PC+Ch (**Figure 5A**). Next we determined whether B-subunits show similar ceramide requirement for binding to Gb3. Binding of Stx B-subunits to Lyso-Gb3+PC+Ch was studied using ELISA. Stx1-B bound equally to both Gb3 and Lyso-Gb3. Unlike the holotxins Stx2a-B also showed similar binding to Lyso-Gb3 and Gb3 (**Figures 3A** **and** **5B**). To explore whether unstable B-pentamer of Stx2a enabled its Lyso-Gb3 binding, we studied Lyso-Gb3 binding of the stabilized B-subunit mutant Stx2a Q40L. To our surprise, the stabilized Q40L also bound to both Gb3 and Lyso-Gb3 (**Figures 3A** **and** **5B**).

## Discussion

Previous reports suggested that B-subunit activities such as receptor binding and toxin internalization play an important role in determining Stx toxicities [28], [49]. Receptor interaction differences of purified toxoids of Stx1 and Stx2a have been previously reported [40]. However, not much information is available about the receptor interactions of Stx2 variants, which significantly differ in toxicity. Here we report, for the first time, the glycolipid receptor binding preferences of holotoxins and B-subunits of Stx2 variants.

Published studies using thin layer chromatography (TLC) overlay with Stx B-subunits or high concentrations of Stx holotoxins have demonstrated that Stx2 binds to Gb3 alone, although less effectively than Stx1 [39]. In our studies, Stx2a shows strong binding to Gb3 (and Gb4) in the presence of PC and Ch (**Figure 3**). Glycan presentation, or the manner in which the glycans are oriented and displayed to the protein, is known to be a critical factor for binding [38]. It is not clear how glycolipids separated by TLC are oriented. However glycolipids immobilized on a hydrophobic microtiter plate likely replicate the two-dimensional display on a biological membrane, where the hydrophobic lipid is attached to the plate and has limited availability compared to the hydrophilic glycans. Thus we believe the glycolipid immobilized on a hydrophobic microtiter plate in our ELISA studies is more likely to resemble Gb3 presentation in the context of a cellular membrane.

The Stx variants displayed distinct glycolipid binding profiles. In most cases the isoforms most toxic to humans, Stx1, Stx2a, Stx2c and Stx2d showed strong glycolipid binding, whereas the weakly toxic form, Stx2b, showed very weak glycolipid binding. This property has diagnostic implications. Capturing Stx by host cell receptors provides a new diagnostic approach to identify and differentiate strains producing Stx variants, which are highly toxic to humans from variants, which are not toxic to humans.

The Bmax for Stx1 binding to Gb3 alone was significantly higher than Stx2a binding to Gb3 alone (**Figure 2** **and** **Table 4**). However the K_D_ values were not very different. It is known that Gb3 binding of Stx2a, but not Stx1, is highly selective [50]. Previous studies demonstrated that cholesterol stabilizes Gb3 in a conformation favorable for binding Stx [40]. It is likely that in the absence of cholesterol, most of the Gb3 does not assume the appropriate conformation to promote binding; however the K_D_ is the same for the few molecule that do assume a conformation favorable for Stx2a binding. As a result saturation is reached with fewer Stx2a molecules, thereby decreasing the Bmax without affecting the K_D_. In support of this hypothesis, similar results were reported with pertussis toxin, another AB_5_ toxin, which displayed different Bmax values for ligands with varying flexibilities, without affecting the K_D_ [51].

Previous studies showed increased Gb3-binding of purified toxoids of Stx1 and Stx2a in the presence of PC and Ch [46]. Hydroxyl group of Ch was shown to improve Stx-Gb3 interaction. Consistently, in our studies presence of PC and Ch improved Gb3 binding of the holotoxin supernatants of all Stx variants compared to Gb3 alone. The Bmax and K_D_ values for most of the Stx2 variants were similar to Stx1 for Gb3+PC+Ch. Published reports suggest that lateral interaction with another glycolipid might improve Gb3 orientation for increased interaction with Stx [38], [52]. In agreement with this we observed Glc-Cer, Gal-Cer and Lac-Cer to improve Stx-Gb3 interaction both in the presence and absence of PC and Ch.

Binding of B-subunits prepared from Stx2a clone was identical to B-subunits prepared from Stx2d clone, consistent with the fact that the amino acid sequences of B-subunits of these variants are identical (**Figure 1**). In contrast, the C-termini of the A-subunits differ for Stx2a and Stx2d. Whereas A-subunit of Stx2a possesses a basic lysine at the C-terminus, Stx2d contains acidic glutamate. Crystal structures of Stx2a and glycan-bound Stx2a suggest that the C-terminus of the A-subunit might take part in receptor recognition. This demonstrates that the slight differences observed between Stx2a and Stx2d holotoxin binding could be due to the A-subunit.

Individual glycan binding sites on Stx display low affinity binding, and host cell recognition is thought to be due to avidity, or the ability to engage several glycan receptors by utilizing multiple binding sites [28], [53], [54]. Consistent with this, the stable Stx1 B-wild type pentamer displayed stronger glycolipid binding than the unstable Stx1 B-subunit mutant L41Q. Similarly, stabilized Stx2a B-subunit mutant Q40L bound better than unstable Stx2a B-Wild type, suggesting that pentamer stability affects receptor binding. This increased binding of the stable B-pentamers is likely due to increased avidity by interaction of all Gb3-binding sites, including the inter-subunit Gb3-binding sites. Previously using AUC, Conrady et al showed that the destabilized Stx1 B-subunit mutant, L41Q, was less stable than Stx1, however more stable than Stx2a B-subunits [46]. Based on this, we had expected the L41Q mutant to show decreased glycolipid affinity than B-subunits of Stx1, but still higher than Stx2a B-subunits. To our surprise, the Stx1 L41Q mutant showed the weakest glycolipid binding of all B-subunits tested (**Figure 4** **and** **Table 4**). This suggests that Stx1 B-subunits are capable of binding to glycolipids only as a stable pentamer. Stx2 B-subunits on the other hand can bind to glycolipids even in lower order oligomeric states.

The Hill coefficients for Gb3 binding of the B-subunits were significantly different. Whereas Stx1 B-subunits bound to Gb3+PC+Ch with a h value of 1 suggesting no cooperativity, Stx2a B-subunits bound with a h value of 2.4 suggesting strong positive cooperativity. Previous studies by AUC showed that at high concentrations (8 µM) Stx2a B-subunits predominantly exist as pentamers, while a small proportion exists in the form of lower order oligomers. On the other hand, predominantly lower order oligomers exist at concentrations lower than 2 µM. Positive binding cooperativity observed with Stx2 B-subunits suggests that binding of these lower order oligomers may occur in two steps, initially B-subunits bind as monomers, and binding of one B-subunit promotes binding of additional B-subunits to form higher order oligomers, ultimately forming pentamers. Since the pentamer formed by Stx1 B-subunits is more stable, this effect is not seen as prominently as with Stx2 B-subunits. Overall, this suggests that the B-subunits of Stx are capable of associating at the glycolipid interface.

Holotoxins of Stx2 variants bound only to the intact glycolipid and no binding was observed to Lyso-Gb3, which lacked carbonyl and a fatty acid chain of Gb3. On the other hand, Stx1 holotoxin and Stx2a B-subunits, irrespective of the pentamer stabilities, did not differentiate between Gb3 and Lyso-Gb3, suggesting that the B-subunits are flexible about fatty acid requirement. Crystal structures of Stx holotoxins show that the C-terminus of A-subunit of Stx2 extends through the pore formed by the B-pentamer and could occlude receptor binding to a region defined as site 3 in Stx1 [14]. Consistently, in the recently reported co-crystal structure only two NAcPk disaccharide densities were reported on the B-subunit of Stx2a holotoxin [44]. It was speculated that the A-subunit interfered with binding to the glycan, which lacked the ceramide. It is therefore possible that the ceramide portion of Gb3 is important for engaging the A-tail of Stx2a thereby opening glycan-binding sites on the B-subunits. Currently we are purifying Stx A-subunits to determine whether the A-subunits are capable of interacting with the glycolipids.

Taken together, this report gives the first account of glycolipid binding preferences of Stx2 variants and the role of B-subunits in these interactions. The knowledge of receptor binding preferences of Stx variants will not only provide understanding of the different toxicities of these highly related variants but it will also provide a means to detect and differentiate these variants during a STEC outbreak.
